# Systemic Granulomatous Diseases Associated with Multiple Palpable Masses That May Involve the Breast: Case Presentation and an Approach to the Differential Diagnosis

**DOI:** 10.1155/2014/146956

**Published:** 2014-09-30

**Authors:** Rodrigo Menezes Jales, Patrick Nunes Pereira, Rafael Fantelli Stelini, Luciano Moro

**Affiliations:** ^1^Department of Obstetrics and Gynecology, Faculty of Medical Sciences, State University of Campinas (Unicamp), P. O. Box 6111, 13083-970 Campinas, SP, Brazil; ^2^Department of Radiology, Faculty of Medical Sciences, State University of Campinas (Unicamp), 13083-970 Campinas, SP, Brazil; ^3^Division of Dermatopathology, Department of Anatomic Pathology, Faculty of Medical Sciences, State University of Campinas (Unicamp), 13083-970 Campinas, SP, Brazil; ^4^Department of Radiology, Faculty of Medicine, University of São Paulo (USP), 05403-900 São Paulo, SP, Brazil

## Abstract

Palpable mass is a common complaint presented to the breast surgeon. It is very uncommon for patients to report breast mass associated with palpable masses in other superficial structures. When these masses are related to systemic granulomatous diseases, the diagnosis and initiation of specific therapy can be challenging. The purpose of this paper is to report a case initially assessed by the breast surgeon and ultimately diagnosed as granulomatous variant of T-cell lymphoma, and discuss the main systemic granulomatous diseases associated with palpable masses involving the breast.

## 1. Introduction

Palpable mass is a common complaint presented to the breast surgeon [1]. It is very uncommon for patients to report breast mass associated with palpable masses in other superficial structures.

Idiopathic granulomatous mastitis is a rare, chronic, autoimmune, benign breast disease, which may mimic breast abscess or malignancy. Although not fully understood, this disease is familiar to the breast surgeon [2]. In contrast, the breast surgeon may not be accustomed to seeing systemic granulomatous diseases. These systemic conditions may initially present as a palpable mass in the breast and other superficial body structures. Causes are varied and include tuberculosis; other mycobacteria or fungi infections; vasculitis, for example, Wegener's granulomatosis and Churg-Strauss syndrome; chronic berylliosis or another metal exposure disease; foreign body reactions; and lymphoma [3]. These conditions may be misdiagnosed and initiation of specific therapy can be challenging [4].

The purpose of this paper is to report a case primarily assessed by the breast surgeon and ultimately diagnosed as granulomatous variant of T-cell lymphoma and discuss the main differential diagnoses of systemic granulomatous diseases associated with palpable masses involving the breast.

## 2. Case Report

A 55-year-old African Brazilian female consulted a breast surgeon at the Breast Division of the Campinas State University Hospital about painless, palpable, bilateral breast masses, first noticed 6 months prior to examination. During this period, she noticed similar masses in the right submandibular region, abdominal wall of the left flank, and left popliteal fossa.

Current complaint included mild exertional dyspnea but there was no fever or weight loss. Past medical history included diabetes mellitus and hypertension, controlled by metformin and hydrochlorothiazide, respectively.

On physical examination, the patient was obese (body mass index: 33.8 Kg/m^2^) with a blood pressure of 130 × 90 mm Hg. Concerning the palpable masses, with the exception of the left breast mass, which was ill-defined, all other masses were circumscribed, fibroelastic, and fixed to deep planes, ranging in size from 2 to 9 cm. There was no skin or nipple retraction. The mass on the abdominal wall was accompanied by skin hyperpigmentation. There was no evidence of regional lymphadenopathy.

Initially, the breast surgeon suspected primary left breast cancer. The breast mass associated with the other masses could be either fortuitous or breast cancer metastasis. The patient was then referred to bilateral diagnostic mammography and ultrasonography for evaluation of breast disease. She was also referred to abdominal, cervical, popliteal, and abdominal wall ultrasonography for assessment of the other masses.

**Figure 1:**
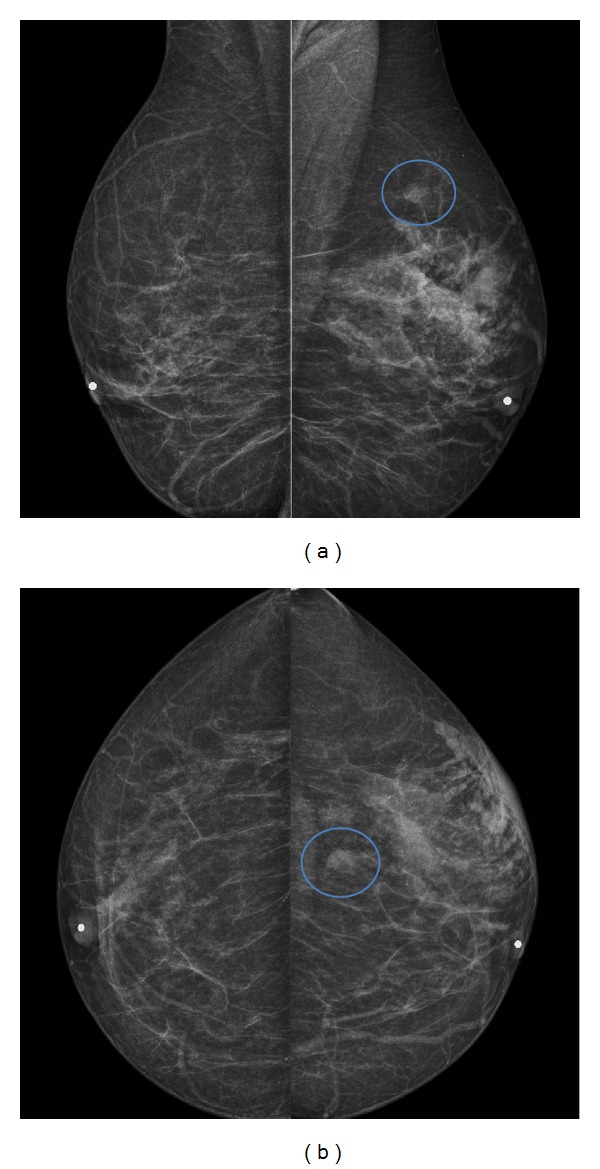
*Bilateral mammography*: bilateral mediolateral oblique (a) and craniocaudal (b) mammographic views demonstrate scattered fibroglandular tissue with global asymmetry and focal skin thickening in the left breast which was classified as moderate suspicious BI-RADS 4c. There was also an isodense, round, circumscribed mass, measuring 12 × 8 mm in the outer upper quadrant, requiring complementary ultrasonography (encircled). Nipples are marked.

Bilateral mediolateral oblique (MLO) and craniocaudal (CC) mammographic views demonstrated scattered fibroglandular tissue with global asymmetry and focal skin thickening in the left breast (arrow), categorized as moderately suspicious (BI-RADS 4c) (Figures 1(a) and 1(b)). There was also an isodense, round, circumscribed mass, measuring 12 × 8 mm in the outer upper quadrant, which required supplemental ultrasonography. The palpable masses in the right breast were quite lower (near the inframammary fold) and not identified on routine incidences.

**Figure 2:**
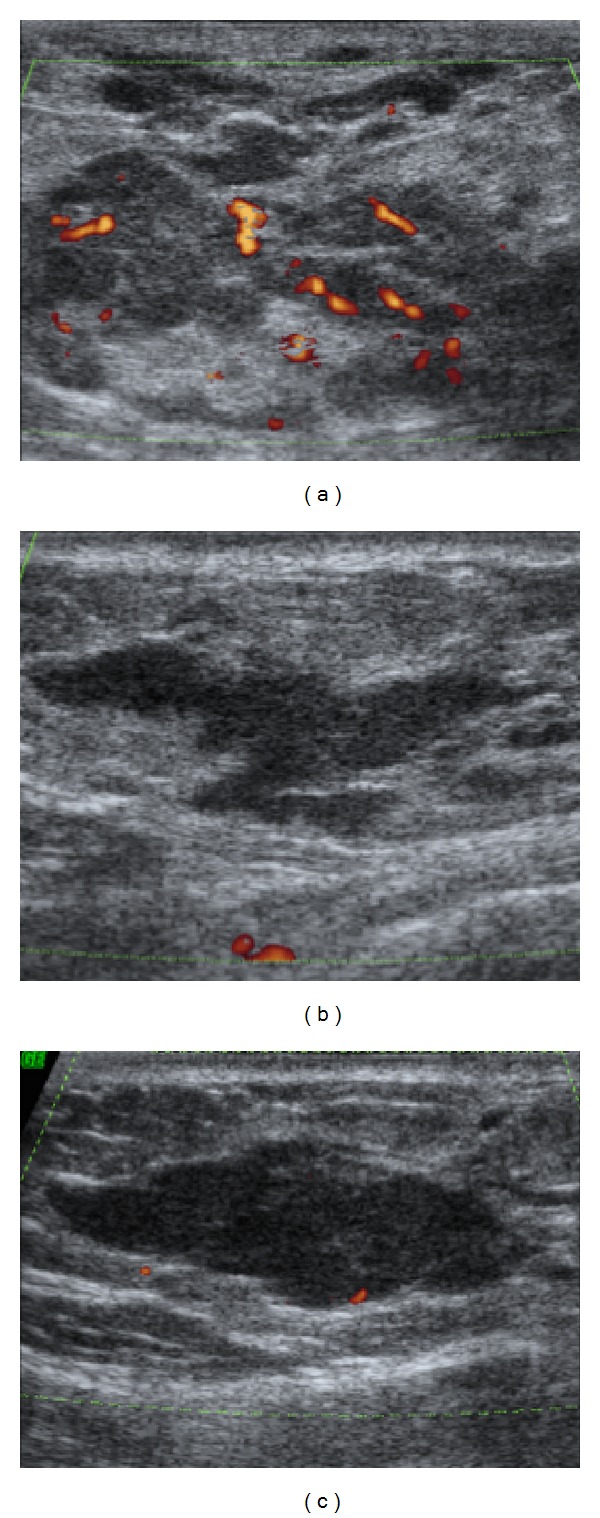
*Breast ultrasonography*: ultrasonography of the left breast confirmed a hypoechoic, irregular, indistinct mass, with internal vascularity, measuring 47 × 16 mm (a). Ultrasonography of the right breast depicted two irregular, hypoechoic, circumscribed, parallel to the skin masses measuring 29 × 9 mm and 17 × 8 mm in the lower outer quadrant, near the inframammary fold (b and c).

Ultrasonography of the upper outer quadrant of the left breast confirmed a hypoechoic, irregular, indistinct mass with internal vascularity, measuring 47 × 16 mm (Figure 2(a)). The circumscribed mass previously identified on left mammography was not recognized on ultrasonography and could be related to an isoechoic mass or intramammary lymph node. Ultrasonography of the lower outer quadrant of the right breast, near the inframammary fold, depicted two irregular, hypoechoic, circumscribed masses parallel to the skin, measuring 29 × 9 and 17 × 8 mm (Figures 2(b) and 2(c)). Breast ultrasonography was also categorized as moderately suspicious (BI-RADS 4c).

**Figure 3:**
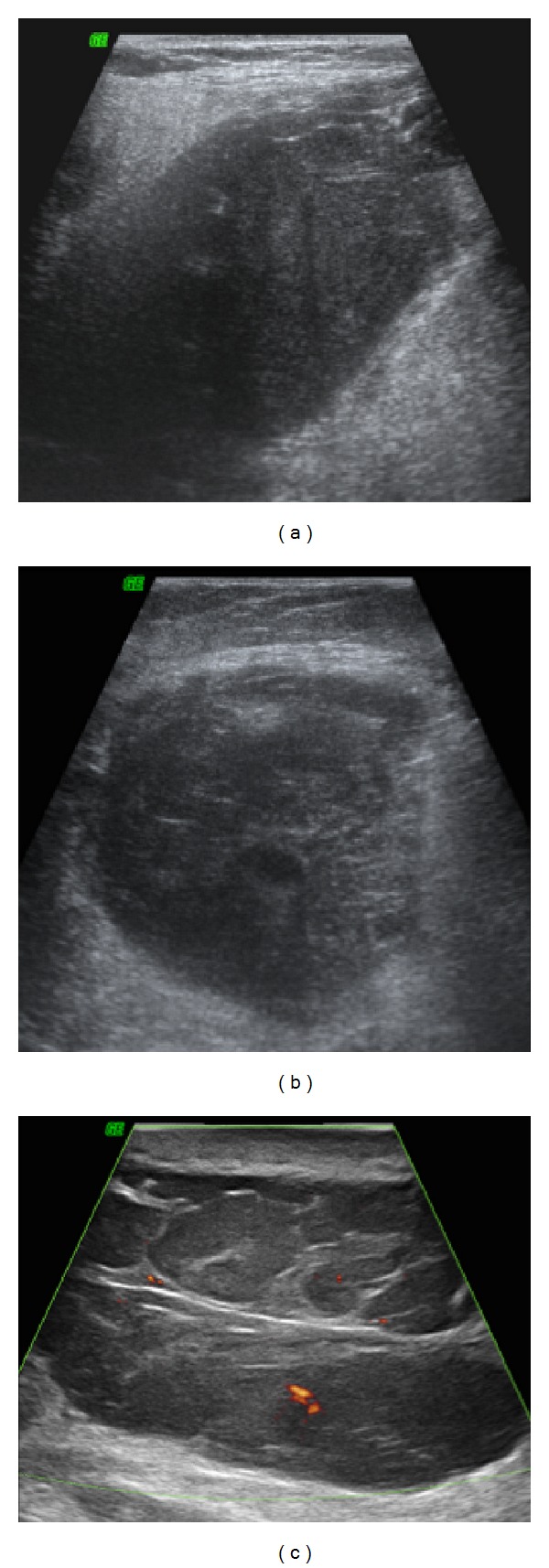
*Small parts ultrasonography*: cervical (a), popliteal (b), and abdominal wall (c) ultrasonography confirmed hypoechoic, well-defined masses in right submandibular gland topography, gastrocnemius muscle thickness, and the subcutaneous tissue, measuring 54 × 37 mm, 98 × 44 mm, and 73 × 47 mm, respectively.

Cervical, popliteal, and abdominal wall ultrasonography confirmed hypoechoic, well-defined masses in the right submandibular gland topography, gastrocnemius muscle thickness, and subcutaneous tissue, respectively. Details of these findings are shown in Figures 3(a), 3(b), and 3(c).

**Figure 4:**
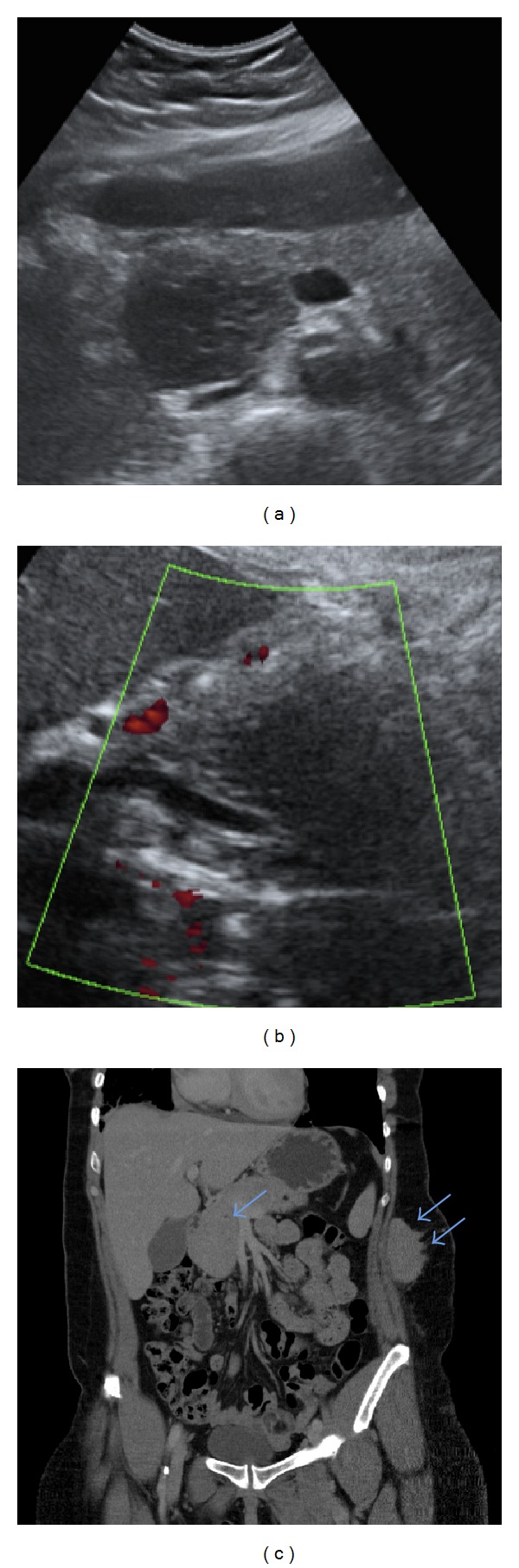
*Abdominal ultrasonography and tomography*: abdominal ultrasonography showed a hypoechoic, ill-defined, pancreatic mass measuring 49 × 36 mm (a). Although the mass affected the pancreatic head, the patient manifested no jaundice. This is justified by the absence of bile duct compression (b). Coronal abdominal CT imaging revealed a solid ill-defined mass in the pancreatic head (single arrow) (c) and another similar lesion in the subcutaneous tissue of the left lateral abdominal wall (double arrows) (c).

Abdominal ultrasonography showed a 49 × 36 mm hypoechoic, ill-defined pancreatic mass (Figure 4(a)). Although the mass affected the pancreatic head, the patient did not manifest jaundice. This is justified by the absence of bile duct compression (Figure 4(b)).

The majority of masses perceived simultaneously by the patient had a similar ultrasonographic appearance, suggesting that most or all lesions could be related to the same condition. In contrast, involvement of different structures, for example, glandular parenchyma, subcutaneous tissue, and muscle fibers, encouraged us to sample each palpable mass to ensure that all were responsible for a single condition. Thus, apart from the submandibular mass, which was evaluated by fine-needle aspiration biopsy (FNAB), the soft tissue and breast masses were investigated by ultrasound-guided percutaneous fragment biopsy with a 14-gauge needle (Bard Biopsy Systems, Tempe, AZ, USA).

Results of FNAB were unsatisfactory per hemorrhagic material. Routine hematoxylin-eosin stained sections of the soft tissue and breast masses, including the left suspicious breast mass, revealed lymphocytic granulomatous eosinophil-rich infiltration. The search for fungal and acid-fast bacilli (AFB) using, respectively, Grocott and Ziehl-Neelsen staining was negative. At that point, the pathology report indicated that inflammatory process was the most likely diagnosis, including sarcoidosis as a possibility, and the breast surgeon referred the patient to the rheumatology outpatient clinic.

On physical examination, the rheumatologist obtained the same findings as those described by the breast surgeon two weeks previously but identified slight exophthalmos associated with right eyelid edema. The patient was admitted to the rheumatology ward for further investigation.

Hematological, clinical chemistry, and radiological investigations were as follows.

Serum chemistry: glucose, C-reactive protein, liver enzymes (alanine aminotransferase, aspartate aminotransferase, and alkaline phosphatase), and kidney function (BUN and creatinine) revealed no abnormalities.

Peripheral blood counts: 6.5 10^3^/mm^3^ total white blood cells (normal range: 4–10); differential white blood cells revealing eosinophilia with 1.5 10^3^ (23.5%) eosinophils (normal range: 0–0.45); 5.2 10^6^/mm^3^ total red blood cells (normal range: 4.2–5.4); 13.8 g/dL hemoglobin (normal range: 12–16); 220 10^3^/mm^3^ platelets (normal range: 150–400).

Abdominal CT confirmed an ultrasonographic mass in the pancreatic head (Figure 4(c)). There were no other masses or lymph node enlargement.

**Figure 5:**
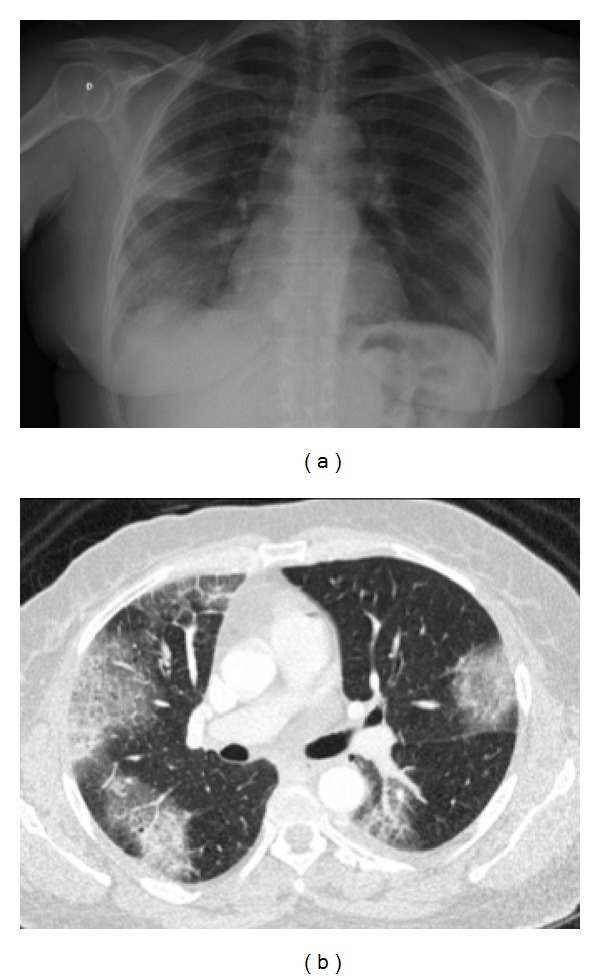
*Chest X-ray and tomography*: chest X-ray revealed ill-defined opacities in both lungs, especially in the middle and inferior field of the right lung (a). There was no mediastinal enlargement, a remarkable sarcoidosis radiographic sign. Axial high-resolution computer tomography (HRCT) scan revealed multiple ground-grass opacities in bilateral subpleural areas, associated with interlobular septal thickening (b).

Chest X-ray revealed ill-defined opacities in both lungs, especially in the middle and inferior field of the right lung. There was no mediastinal enlargement, which is a remarkable sarcoidosis radiographic sign (Figure 5(a)). A chest high-resolution computed tomography (HRCT) showed multiple ground-glass opacities in subpleural areas of both lungs, associated with interlobular septal thickening (Figure 5(b)). Tuberculin skin test was negative.

**Figure 6:**
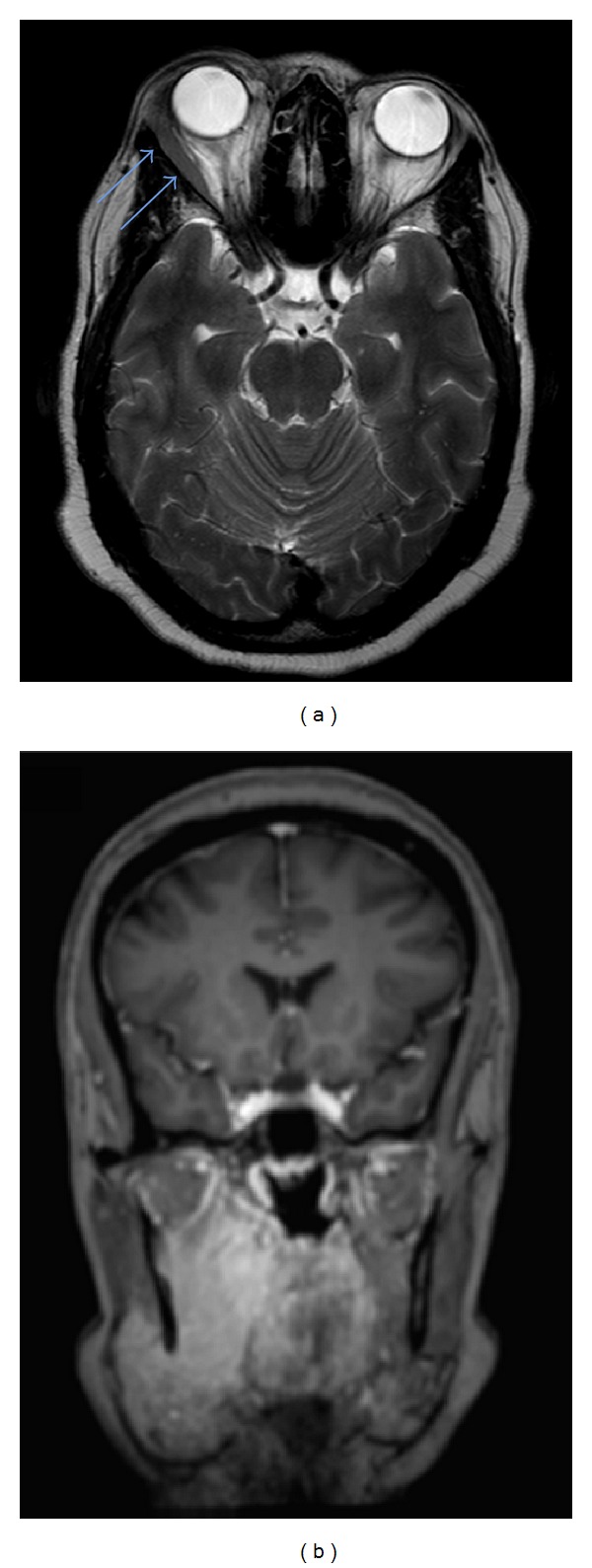
*Head and neck magnetic resonance imaging*: axial T2 MRI revealed enlargement of the lateral rectus muscle of right orbit (arrows) (a). A coronal contrast-enhanced T1 MRI showed an infiltrating lesion centered in the right masticator space extending into the submandibular space involving the submandibular gland. Note the avid gadolinium enhancement pattern of the lesion and its ill-defined margins (b).

Magnetic resonance imaging (MRI) of the brain was within normal limits, except for enlargement of the lateral rectus muscle of the right orbit, justifying clinical exophthalmos (Figure 6(a)). Head and neck MRI showed an infiltrative lesion centered in the right masticator space that extended into the submandibular space involving the submandibular gland with avid gadolinium enhancement pattern and ill-defined margins (Figure 6(b)).

Owing to the lack of mediastinal lymph node enlargement, which is a remarkable sarcoidosis radiographic sign, bronchoscopy with biopsy was ordered. The exam was considered normal. Lung biopsy also showed diffuse interstitial granulomatous chronic inflammation, with a small amount of eosinophils. The search for fungal and acid-fast bacilli (AFB) was negative. Microscopy and acid-fast bacilli (AFB) culture of bronchoalveolar lavage fluid also yielded negative results. The pneumopathologist indicated that histologic findings may correspond to Churg-Strauss syndrome.

**Figure 7:**
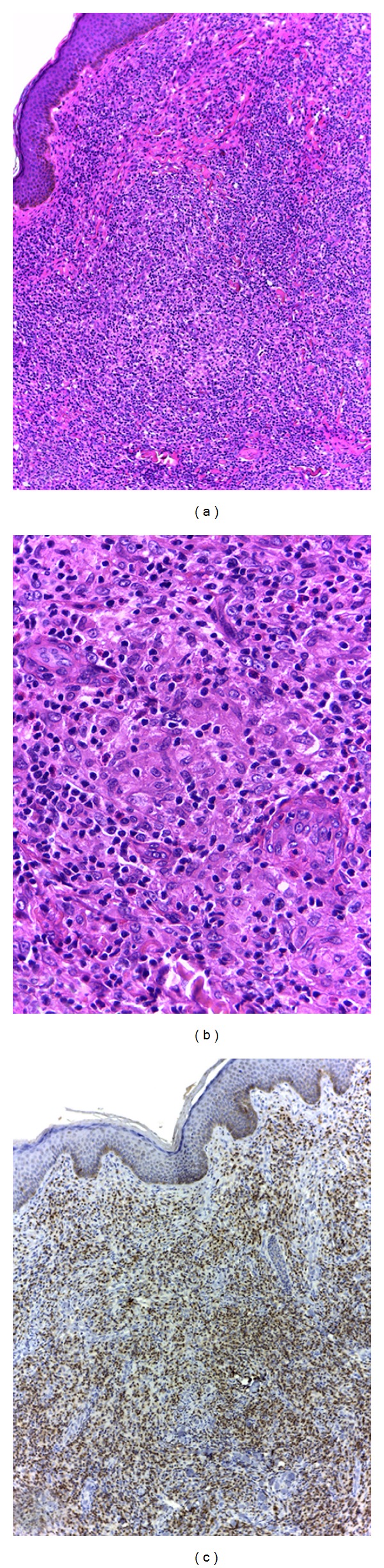
*Pathological diagnosis*: routine hematoxylin-eosin stained section of the skin of abdominal wall (a and b) revealed lymphocytic and granulomatous infiltration, with epithelioid macrophages and sparse eosinophils (original magnification: [100x] and [400x], resp.). Immunohistochemical study (c) showed positivity for CD3, a T-cell marker (original magnification: [100x]).

**Figure 8:**
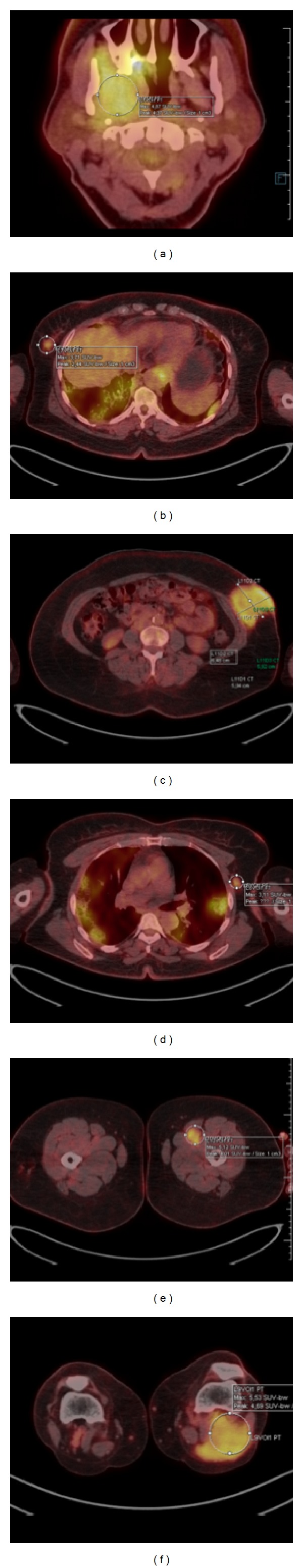
*Positron emission tomography (PET-CT)*: there were hypermetabolic lesions at previously known locations, such as the right masticator space (a), right breast (b), left abdominal wall (c), and left popliteal fossa (f), and also locations that were not previously diagnosed such as left axillary lymph node (d) and the left thigh muscle (e).

Incisional biopsy of a larger sample of the abdominal wall skin mass under local anesthesia was suggested in a multidisciplinary clinical meeting, since it was an accessible site. The skin specimen showed a monotonous lymphocytic infiltration, intermingled by irregular granulomas, and frequent eosinophils, occupying entirely the dermis (Figures 7(a) and 7(b)). Lymphocytes were diffusely positive for T-cell markers (CD2, CD3, CD5, and CD7) (Figure 7(c)). Search for fungal and AFB was negative. The histopathologic diagnosis of granulomatous T-cell non-Hodgkin lymphoma in skin of the abdomen was rendered. Patient referral to the hematology ward was required. Histologic findings in association with patient's medical history were considered consistent with peripheral T-cell extranodal non-Hodgkin lymphoma. The hematologist sent the patient to PET-CT for lymphoma staging (Figures 8(a), 8(b), 8(c), 8(d), and 8(e)). There were hypermetabolic lesions in previously known sites and also in cervical, axillary, and paravertebral lymph nodes.

**Figure 9:**
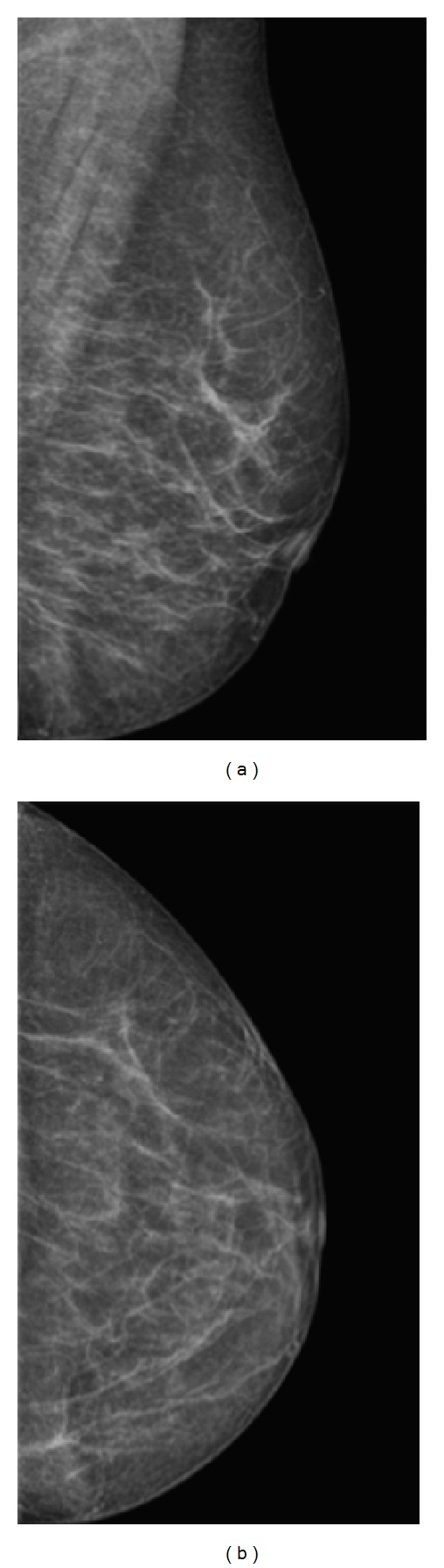
*Posttreatment mammography*: left breast mediolateral oblique (a) and craniocaudal (b) mammographic views confirmed complete left breast lesion resolution after the 3rd chemotherapy cycle.

Six chemotherapy cycles with CHOEP (Adriamycin/doxorubicin, cyclophosphamide, vincristine/Oncovin, and etoposide/Vepesid) were indicated. After 3 chemotherapy cycles, there was resolution of right exophthalmos and the mass was no longer clinically palpable. Mammography confirmed breast lesion resolution (Figures 9(a) and 9(b)). However, after the 6th chemotherapy cycle, there were still residual hypermetabolic lesions in the lung and popliteal fossae identified by PET-CT. The treatment will be now supplemented with bone marrow transplantation.

## 3. Discussion

The granulomatous inflammatory reaction is usual in pathology, being a manifestation of many infective, toxic, allergic, autoimmune, and neoplastic diseases [5]. Despite being a specific inflammatory pattern, it is not a specific histologic finding of a particular etiology. Pathology findings, even including immunohistochemical techniques, may not be useful enough to achieve a definitive diagnosis and state proper therapy.

In this paper we discuss the main systemic granulomatous diseases that may affect the breasts and how they were related to the reported case.

### 3.1. Tuberculosis

About one-third of the world's population has latent* Mycobacterium tuberculosis* infection. There has been a significant rise in the prevalence of nontuberculous and extrapulmonary mycobacterium infection due to an aging population, AIDS-related opportunistic infection, and immigration from tuberculosis-endemic regions [6, 7]. Our university is located in a country where pulmonary tuberculosis is still endemic and tuberculosis infection is a hypothesis that should always be considered [8, 9].

### 3.2. Breast Involvement

The breast and other palpable surfaces can be primary sites of tuberculosis (TB) infection. However,* Mycobacterium tuberculosis* infection represents only up to 4% of all breast lesions in countries where TB is endemic [10, 11]. The infection most commonly reaches the breast through lymphatic spread from the axillary, mediastinal, or cervical nodes or directly from underlying structures such as the ribs [12, 13].

Due to nonspecific clinical and radiological findings, it is difficult to make the diagnosis of breast TB [10]. Breast compromise is usually unilateral, but it may be bilateral [14]. Clinical presentation may include acute abscess, a round, slowly growing nodular mass, or even a lesion strikingly similar to breast carcinoma [14]. An associated axillary lymphadenopathy may be found [12]. Furthermore, breast TB is paucibacillary. As a result, the tuberculin skin test, microscopy, culture, and nucleic acid amplification techniques do not have the same diagnostic utility as in pulmonary tuberculosis [15].

Therefore, TB infection of the breast and other extrapulmonary sites may be misdiagnosed as another granulomatous disease [16]. A granulomatous extrapulmonary condition that fails to respond to specific therapy should give rise to the suspicion of culture-negative tuberculosis. In the majority of cases, the final diagnosis is only possible with a high index of suspicion [16].

### 3.3. Sarcoidosis

Sarcoidosis is an enigmatic granulomatous systemic disease and its etiology has not been clearly elucidated [17]. Sarcoid granulomas can involve any organ, but more than 90% of patients present with intrathoracic lymph node enlargement, cutaneous or ocular signs and symptoms, and pulmonary involvement. Pulmonary compromise is the predominant clinical feature [18]. Ocular disease occurs in approximately one-third of sarcoidosis patients. [19]. Occasionally, the clinical presentation of sarcoidosis includes signs and symptoms involving the head and neck [20]. It is usually a diagnosis of exclusion that is based on compatible clinical/radiological findings and supported by histologic evidence in one or more organs.

Classic chest radiographic imaging, either as an initial workup of respiratory symptoms or as an incidental finding, may reveal bilateral hilar lymphadenopathy and/or reticular parenchymal opacities [19]. High-resolution chest CT may reveal a variety of additional abnormalities, including nodularity, bronchial wall thickening, and stranding predominantly in the middle and apical regions of the lungs [21]. These findings were not clear on chest imaging of the case described.

A biopsy specimen should be obtained from the compromised organ that is most easily accessed [17]. In the case described, because of the different structures involved, for example, the glandular parenchyma, subcutaneous tissue, and muscle, we chose to sample each palpable mass to ensure that all accounted for a single condition. Sarcoidosis may mimic or occur concomitantly with malignancy [22]. Furthermore, since sarcoidosis is a diagnosis of exclusion, the physician should always maintain a healthy degree of skepticism concerning the diagnosis [3]. On the other hand, due to lack of specificity of granulomatous diseases and the continued high prevalence of tuberculosis in our country, many sarcoidosis patients are misdiagnosed with tuberculosis and treated for the condition [23].

### 3.4. Breast Involvement

Sarcoidosis of the breast occurs in less than 1% of sarcoidosis patients [24]. The majority of cases occur in patients who were previously diagnosed with sarcoidosis in other anatomic sites. Thus, breast mass as the primary manifestation of sarcoidosis is extremely rare [25].

There has been no description of uniform clinical findings [26–28]. However, lesions may be fixed or demonstrate skin dimpling and peau d'orange appearance resembling carcinoma [22, 25]. Thus, breast sarcoidosis on mammography and ultrasonography may also mimic malignancy, including enlarged axillary lymph nodes [22, 29].

### 3.5. Churg-Strauss Syndrome

Eosinophilic granulomatosis with polyangiitis or Churg-Strauss syndrome is a systemic granulomatous small-vessel vasculitis associated with eosinophilia [30, 31]. Asthma is a well-established and prominent clinical hallmark [30–32]. The precise mechanism of pathogenesis has only been partly elucidated [31]. The syndrome commonly manifests with upper airway tract and lung involvement, peripheral neuropathy, and cardiac and skin lesions [33]. Cardiac involvement is the major cause of early death and poor long-term prognosis [34, 35]. Migratory infiltrates visualized on chest radiography are a key feature of Churg-Strauss syndrome but only 64% of patients had abnormal findings on chest X-rays [36].

In the present case, although eosinophilia was in agreement with the diagnosis of Churg-Strauss, the absence of clinical symptoms such as asthma or neuropathy was inconsistent with this syndrome.

### 3.6. Breast Involvement

Although rare, breast vasculitis may be an isolated finding or a manifestation of systemic vasculitis [37]. The clinical appearance often resembles malignancy but a dramatic response to steroid therapy can be expected [37, 38].

The early phase of Churg-Strauss syndrome is characterized by extravascular eosinophilic tissue infiltration in virtually any organ. However, breast involvement is extremely rare [31, 39]. It has been previously reported that masses and mastitis are manifestations of breast compromise [38, 40].

### 3.7. Lymphoma

Lymphoma is a large group of lymphocyte cancers. There are two basic categories of lymphomas: Hodgkin and non-Hodgkin lymphoma. Hodgkin lymphoma is marked by the presence of the Reed-Sternberg cell. Non-Hodgkin lymphoma includes a large, diverse group of cancers. Prognosis and treatment depend on the lymphoma type and stage [41]. Immunohistochemical study plays a key role in the classification and subclassification of lymphomas by detection of lineage-specific antigens [42].

The occurrence of epithelioid cell granuloma is associated with many neoplasms, including lymphoma, which may cause difficulties with interpretation and delay in making the final diagnosis [43].

### 3.8. Breast Involvement

Primary breast lymphomas usually account for <1% of all non-Hodgkin lymphomas and malignant breast neoplasms [44]. The low prevalence may be related to the very small amount of lymphoid tissue contained in the breast [45].

Breast lymphoma may occur either as a primary or as a secondary lesion. For the diagnosis of primary breast lymphoma, it has been proposed that the breast should be the first or major site of disease presentation. There should be no evidence of lymphoma elsewhere, except for the ipsilateral axillary node [46]. Secondary breast lymphoma, as in the case described, is more common [47]. On the other hand, it was found that 94% of breast lymphomas were of B-cell lineage and only 6% were of T-cell type, as in the case reported [47].

The most common symptom of breast lymphoma is a painless, palpable mass. Nipple retraction or discharge and skin changes can also rarely occur [44, 47]. Radiological findings associated with other breast malignancies, such as calcifications, spiculations, or architectural distortions, are extremely rare [45].

The majority of B-cell breast lymphomas occur as palpable masses, whereas skin changes, edema, and local tenderness are more commonly associated with T-cell lymphomas [45, 48]. Ipsilateral axillary lymphadenopathy has been reported in 13% to 50% of cases [49].

The diagnosis and classification of lymphomas may depend on clinical-pathological correlation. This is of paramount importance since a breast mass containing lymphoma cells does not require excision, in contrast to breast carcinoma. Therefore, to avoid unnecessary treatment, the breast surgeon must be aware of breast lymphoma, especially when atypical clinical and radiological findings are encountered.

## 4. Conclusion

The report of palpable masses in both the breast and other superficial structures may be related to rare systemic granulomatous diseases. Minimal invasive diagnosis (e.g., core needle biopsy) is essential to guide the process of differential diagnosis. However, a multidisciplinary approach including breast surgeon, rheumatologist, hematologist, infectious disease specialist, dermatologist, pathologist, and radiologist may be necessary to achieve a definitive diagnosis and proper treatment.

## References

[1] Klein S (2005). Evaluation of palpable breast masses. *American Family Physician*.

[2] Poovamma CU, Pais VA, Dolas SC, Prema M, Khandelwal R, Nisheena R (2014). Idiopathic granulomatous mastitis: a rare entity with a variable presentation. *Breast Diseases*.

[3] Judson MA (2007). The management of sarcoidosis by the primary care physician. *The American Journal of Medicine*.

[4] Lanng C, Eriksen BØ, Hoffmann J (2004). Lipoma of the breast: a diagnostic dilemma. *Breast*.

[5] Williams GT, Williams WJ (1983). Granulomatous inflammation: a review. *Journal of Clinical Pathology*.

[6] Tewari M, Shukla HS (2005). Breast tuberculosis: diagnosis, clinical features and management. *Indian Journal of Medical Research*.

[7] Harris SH, Khan MA, Khan R, Haque F, Syed A, Ansari MM (2006). Mammary tuberculosis: analysis of thirty-eight patients. *ANZ Journal of Surgery*.

[8] Gemal A, Keravec J, Menezes A, Trajman A (2013). Can Brazil play a more important role in global tuberculosis drug production? An assessment of current capacity and challenges. *BMC Public Health*.

[9] Kakkar S, Kapila K, Singh MK, Verma K (2000). Tuberculosis of the breast. A cytomorphologic study. *Acta Cytologica*.

[10] Akcakaya A, Eryilmaz R, Sahin M, Ozkan OV (2005). Tuberculosis of the breast. *The Breast Journal*.

[11] Tewari M, Shukla HS (2005). Breast tuberculosis: Diagnosis, clinical features & management. *Indian Journal of Medical Research*.

[12] Khanna R, Prasanna GV, Gupta P, Kumar M, Khanna S, Khanna AK (2002). Mammary tuberculosis: report on 52 cases. *Postgraduate Medical Journal*.

[13] Keum DY, Kim JB, Park CK (2012). Surgical treatment of a tuberculous abscess of the chest wall. *Korean Journal of Thoracic and Cardiovascular Surgery*.

[14] van Keirsbilck J, Riphagen I, Struyven H (2008). Bilateral mammary tuberculosis associated with a borderline ovarian tumor. *Breast Care*.

[15] Pai M, Riley LW, Colford JM (2004). Interferon-*γ* assays in the immunodiagnosis of tuberculosis: a systematic review. *The Lancet Infectious Diseases*.

[16] Sriram KB, Moffatt D, Stapledon R (2008). Tuberculosis infection of the breast mistaken for granulomatous mastitis: a case report. *Cases Journal*.

[17] Iannuzzi MC, Fontana JR (2011). Sarcoidosis: Clinical presentation, immunopathogenesis, and therapeutics. *Journal of the American Medical Association*.

[18] Haroon M, Ryan JG, Harney S (2011). Development of sarcoidosis 6-month post discontinuation of etanercept: coincidence or real association?. *Clinical Rheumatology*.

[19] Baughman RP, Lower EE, Kaufman AH (2010). Ocular sarcoidosis. *Seminars in Respiratory and Critical Care Medicine*.

[20] Christoforidou A, Goudakos J, Bobos M, Lefkaditis E, Vital V, Markou K (2013). Sarcoidosis-like granulomatosis of the hypopharynx as a complication of anti-TNF therapy. *The American Journal of Otolaryngology—Head and Neck Medicine and Surgery*.

[21] Müller NL, Mawson JB, Mathieson JR, Abboud R, Ostrow DN, Champion P (1989). Sarcoidosis: correlation of extent of disease at CT with clinical, functional, and radiographic findings. *Radiology*.

[22] Isley LM, Cluver AR, Leddy RJ, Baker MK (2012). Primary sarcoid of the breast with incidental malignancy. *Journal of Clinical Imaging Science*.

[23] Rodrigues MM, Coletta ENAM, Ferreira RG, Pereira CADC (2013). Delayed diagnosis of sarcoidosis is common in Brazil. *Jornal Brasileiro de Pneumologia*.

[24] Hermann G, Nagi C, Mester J, Tierstein A (2008). Unusual presentation of sarcoidosis of the breast. *British Journal of Radiology*.

[25] Ojeda H, Sardi A, Totoonchie A (2000). Sarcoidosis of the breast: implications for the general surgeon. *The American Surgeon*.

[26] Gansler TS, Wheeler JE (1984). Mammary sarcoidosis. Two cases and literature review. *Archives of Pathology and Laboratory Medicine*.

[27] Ross MJ, Merino MJ (1985). Sarcoidosis of the breast. *Human Pathology*.

[28] Wilhelm K, Teifke A, Muller-Quernheim J, Mitze M (1994). Extrapulmonary manifestation of sarcoidosis in the mammary gland. *Aktuelle Radiologie*.

[29] Sabaté JM, Clotet M, Gómez A, de Las Heras P, Torrubia S, Salinas T (2005). Radiologic evaluation of uncommon inflammatory and reactive breast disorders. *Radiographics*.

[30] Vaglio A, Buzio C, Zwerina J (2013). Eosinophilic granulomatosis with polyangiitis (Churg-Strauss): state of the art. *Allergy*.

[31] Mahr A, Moosig F, Neumann T (2014). Eosinophilic granulomatosis with polyangiitis (Churg-Strauss): evolutions in classification, etiopathogenesis, assessment and management. *Current Opinion in Rheumatology*.

[32] Pagnoux C, Guilpain P, Guillevin L (2007). Churg-strauss syndrome. *Current Opinion in Rheumatology*.

[33] Szczeklik W, Grzanka P, Mastalerz L, Sokoåowska B, Musial J (2010). Lung involvement in Churg-Strauss syndrome as related to the activity of the disease. *Allergy*.

[34] Bourgarit A, Le Toumelin P, Pagnoux C (2005). Deaths occurring during the first year after treatment onset for polyarteritis nodosa, microscopic polyangiitis, and Churg-Strauss syndrome: a retrospective analysis of causes and factors predictive of mortality based on 595 patients. *Medicine*.

[35] Guillevin L, Pagnoux C, Seror R, Mahr A, Mouthon L, Toumelin PL (2011). The five-factor score revisited: assessment of prognoses of systemic necrotizing vasculitides based on the french vasculitis study group (FVSG) cohort. *Medicine*.

[36] Furuiye M, Yoshimura N, Kobayashi A (2010). Churg-strauss syndrome versus chronic eosinophilic pneumonia on high-resolution computed tomographic findings. *Journal of Computer Assisted Tomography*.

[37] Allende DS, Booth CN (2009). Wegener's granulomatosis of the breast: a rare entity with daily clinical relevance. *Annals of Diagnostic Pathology*.

[38] Villalba-Nuño V, Sabaté JM, Gómez A (2002). Churg-Strauss syndrome involving the breast: a rare cause of eosinophilic mastitis. *European Radiology*.

[39] Atili A, Richter C, Bahn E, Rustenbeck HH, Schittkowski M (2013). Bereich Strabologie und Neuroophthalmologie, Augen-Praxis-Klink Esslingen. [Ocular manifestations of Churg-Strauss syndrome: review article and case report]. *Ophthalmologe*.

[40] Ben Dhaou B, Derbali F, Boussema F (2012). Eosinophilic nodule of the breast: a rare manifestation in the Churg-Strauss syndrome. *La Tunisie Médicale*.

[41] National Cancer Institute Definition of lymphoma. http://www.cancer.gov/dictionary?cdrid=45368.

[42] Batrani M, Bhawan J (2014). Pitfalls in the diagnosis of cutaneous lymphoma. *The American Journal of Dermatopathology*.

[43] Basu D, Bundele M (2005). Angioimmunoblastic T-cell lymphoma obscured by concomitant florid epithelioid cell granulomatous reaction—a case report. *Indian Journal of Pathology and Microbiology*.

[44] Domchek SM, Hecht JL, Fleming MD, Pinkus GS, Canellos GP (2002). Lymphomas of the breast: primary and secondary involvement. *Cancer*.

[45] Shim E, Song SE, Seo BK, Kim Y-S, Son GS (2013). Lymphoma affecting the breast: a pictorial review of multimodal imaging findings. *Journal of Breast Cancer*.

[46] Wiseman C, Liao KT (1972). Primary lymphoma of the breast.. *Cancer*.

[47] Surov A, Holzhausen HJ, Wienke A (2012). Primary and secondary breast lymphoma: prevalence, clinical signs and radiological features. *British Journal of Radiology*.

[48] Gualco G, Chioato L, Harrington WJ, Weiss LM, Bacchi CE (2009). Primary and secondary T-cell lymphomas of the breast: clinico-pathologic features of 11 cases. *Applied Immunohistochemistry and Molecular Morphology*.

[49] Talwalkar SS, Miranda RN, Valbuena JR, Routbort MJ, Martin AW, Medeiros LJ (2008). Lymphomas involving the breast: a study of 106 cases comparing localized and disseminated neoplasms. *The American Journal of Surgical Pathology*.

